# A scoping review of considerations and practices for benefit sharing in biobanking

**DOI:** 10.1186/s12910-021-00671-x

**Published:** 2021-07-27

**Authors:** Allan Sudoi, Jantina De Vries, Dorcas Kamuya

**Affiliations:** 1 Department of Health Systems and Research Ethics (HSRE), KEMRI-Wellcome Trust Research Programme, KEMRI Centre for Geographic Medicine, Coast, P.O. Box 230-80108, Kilifi, Kenya; 2 Department of Medicine, Faculty of Health Sciences, University of Cape Town, Cape Town, South Africa

**Keywords:** Benefits, Benefit sharing, Biobanking, Biobanks, Ethics, Sample sharing

## Abstract

**Background:**

Despite the rapid global growth of biobanking over the last few decades, and their potential for the advancement of health research, considerations specific to the sharing of benefits that accrue from biobanks have received little attention. Questions such as the types and range of benefits that can arise in biobanking, who should be entitled to those benefits, when they should be provided, by whom and in what form remain mostly unanswered. We conducted a scoping review to describe benefit sharing considerations and practices in biobanking in order to inform current and future policy and practice.

**Methods:**

Drawing on the Arksey and O’Malley framework, we conducted a scoping review of the literature in three online databases (PubMed, Cochrane library, and Google Scholar). We extracted and charted data to capture general characteristics, definitions and examples of benefits and benefit sharing, justification for benefit sharing, challenges in benefit sharing, governance mechanisms as well as proposed benefit sharing mechanisms.

**Results:**

29 articles published between 1999 and 2020 met the inclusion criteria for the study. The articles included 5 empirical and 24 non-empirical studies. Only 12 articles discussed benefit sharing as a stand-alone subject, while the remaining 17 integrated a discussion of benefits as one issue amongst others. Major benefit sharing challenges in biobanking were found to be those associated with uncertainties around the future use of samples and in resultant benefits.

**Conclusion:**

Most of the benefit sharing definitions and approaches currently in use for biobanking are similar to those used in health research. These approaches may not recognise the distinct features of biobanking, specifically relating to uncertainties associated with the sharing and re-use of samples. We therefore support approaches that allow decisions about benefit sharing to be made progressively once it is apparent who samples are to be shared with, the intended purpose and expected benefits. We also highlight gaps in key areas informing benefit sharing in biobanking and draw attention to the need for further empirical research.

**Supplementary Information:**

The online version contains supplementary material available at 10.1186/s12910-021-00671-x.

## Introduction

Biobanking refers to the storage, active sharing and re-use of biological specimens and associated data for research purposes [1, 2]. These specimens, collected specifically for biobanking or leftover from primary research or healthcare, support a wide range of research activities including basic, experimental and clinical research, as well as research applied in the development of tools for prevention, diagnosis and treatment of diseases; including personalized medicine [2, 3]. With increasing research activity and demand for biospecimens for research, the number of biobanks worldwide has increased significantly between 1980 and 1999 [4] with close to 70% of the world’s biobanks located in Europe [4]. Widely discussed ethical issues in literature include: nature of consent (broad, restricted, tiered); who can give informed consent; information to be contained in consent forms [3, 5–7]; privacy and confidentiality issues [3, 8]; ownership of samples [7]; the role of regulation in biobanking [9, 10] among others. An existing specific gap is in the understanding of the types of benefits and benefit sharing frameworks that should guide biobanking.

Schroeder (2007) notes that the concept of benefit sharing has not been well defined and provides a general definition for use with genetic resources; “the action of giving a portion of advantages/profits derived from the use of human genetic resources to the resource providers to achieve justice in exchange, with a particular emphasis on the clear provision of benefits to those who may lack reasonable access to resulting healthcare products and services without providing unethical inducements” [11]. In this definition, the terms ‘advantages/profits’’ are used deliberately to capture the notion that benefit sharing relates to both monetary and non-monetary benefits. Although several studies have explored benefit sharing in health research [11–17] and described benefit sharing frameworks [12, 18, 19], there is little consideration for benefit sharing in biobanking. There are significant debates in health research literature on benefit-sharing including in relation to the different forms of benefits (monetary, health care, infrastructure development, gifts etc.); who should receive the benefits (participants, communities, researchers, the ‘public’); who is responsible for their provision (researchers, sponsors, relevant government bodies, industry) and when the benefits should be provided (framed on a continuum of time of during and after the research) [17]. There are also important questions about how decisions about benefit sharing are made at institutional, national and supra-national levels. Yet these discourses, on nature of benefits and benefit-sharing mechanisms, have rarely been applied to the context of biobanking, leaving important questions about how benefit sharing should – and could – be considered in the context of biobanking. Thus, we undertook a scoping review of existing literature in order to explore further some of these issues, particularly the types and forms of benefits and benefit sharing considerations for biobanking.

## Methods

The scoping review team comprised 3 authors who have varied experience in bioethics, biobanking and genomic research as proposed by Levac and colleagues [20]. The team discussed and agreed on the design of the scoping review including the research question, the search criteria, the databases to use and analytical approach. This scoping review was carried out according to the Arksey and O’Malley framework [21] improved upon by Levac et al. [20]. The review included the following five key phases: (1) identifying the research question, (2) identifying relevant literature, (3) literature selection, (4) charting the data, and (5) collating, summarizing, and reporting the results. The optional ‘consultation exercise’ recommended in the framework was not carried out.

The research question guiding this review was: what are the benefit sharing considerations and practices in biobanking described in literature? More specific questions were: (1) How are benefits and benefit sharing defined with relation to biobanking?; (2) Who are the stakeholders involved in biobanking and how are decisions on benefit sharing made?; (3) what are the motivators, barriers and enablers to benefit sharing in biobanking; and (4) what benefit sharing mechanisms have been proposed?

### Data sources and search strategy

We conducted the initial literature search in September 2020 in three electronic databases, PubMed, Cochrane Library and Google Scholar. The databases were chosen due to their free access, comprehensiveness and were known to cover health-related matters. Google Scholar was included to cater for forms of literature that could not be obtained in the other databases. The search query consisted of terms that covered the two key areas of “benefit sharing” and “biobanking” and was expanded by use of Medical Subject Headings (MeSH) and relevant synonyms. The search was limited to the titles and abstracts of the articles within the databases. In keeping with the suggestion by Bramer et al. to include opposites of key search terms to avoid bias, we also included ‘risk sharing’ within the search criteria [22]. Table 1 describes the search terms used for the PubMed database, with the search query tailored to the specific requirements of each database. For Google Scholar, slightly different search strings were used because of the lack of MeSH terms within the database (see Additional file 2: Appendix 2). In addition to the search strategy described above, we also used the “cited by” function in Google Scholar and examined the reference lists of all the selected articles to identify additional articles that met our inclusion criteria. No date limits were placed on the database search and only articles in English were considered.

**Table 1:** PubMed search criteria

Search terms	Search details
Biobanks/Biobanking	biological specimen banks [MeSH] OR biobank*[tiab] OR biorepositor*[tiab]
Benefits/Benefit sharing	"beneficence"[Mesh] OR "benefit"[tiab] OR "benefit sharing"[tiab] OR "social value" OR "benefit distribution"[tiab]
Risk/Burden sharing	"risk assessment"[Mesh] OR "risk distribution"[tiab] OR "risk sharing"[tiab] OR "burden distribution"[tiab] OR "burden sharing"[tiab]
Final Query	((biological specimen banks[MeSH] OR biobank*[tiab] OR biorepositor*[tiab]) AND ("beneficence"[Mesh] OR "benefit"[tiab] OR "benefit sharing"[tiab] OR "social value" OR "benefit distribution" [tiab])) OR ((biological specimen banks[MeSH] OR biobank*[tiab] OR biorepositor*[tiab]) AND ("risk assessment"[Mesh] OR "risk distribution"[tiab] OR "risk sharing"[tiab] OR "burden distribution"[tiab] OR "burden sharing"[tiab]))

### Citation management

All the citations from the different databases were exported to EndNote X9 and duplicates removed. This was followed by screening for relevant papers guided by a title and abstract screening tool (Additional file 1: Appendix 1).

### Eligibility criteria

Any articles that described any aspect of biobanking and benefit sharing, allocation or distribution was included. Articles were not limited to geographical location and included peer reviewed journal publications, commentaries, editorials and reports. Any articles about benefit sharing not directly related to human health research were removed. Articles about financial banking, banking of animal tissue, banking of microorganisms (e.g. virus or microbiome archives), temporary banking of amputated parts, banking of tissue/organs for care and milk banks for dietary supplementation were excluded because they were out of the scope of the current review.

### Title and abstract relevance screening

Only the titles and abstracts of citations identified in the database search were reviewed during the first stage of screening. Articles of which titles seemed to meet the inclusion criteria but where abstracts were missing were included for full article review in the data characterization phase. AS screened all the articles and the final list of selected articles was then reviewed by JDV and DK. Throughout the screening process AS, JDV and DK met to discuss and resolve any uncertainties related to study selection [20].

### Data characterization

The full articles for all the citations deemed relevant via title and abstract screening and those missing abstracts were obtained for subsequent full text review. For articles that were not openly available, or those that could not be obtained through institutional library access, attempts were made to contact the author for assistance in obtaining them. Any articles that could not be obtained through these processes were excluded. Two separate templates were developed for data abstraction and characterization. The first was a Microsoft Excel sheet that captured study characteristics such as author name(s), publication year, publication type, geographic setting, and area of focus (see Table 2 below). The second was a coding framework developed in NVivo to capture the actual study content that related to the study questions. Both data charting forms (Excel and NVivo) were discussed extensively during analysis by the study team. The characteristics of each full-text article were extracted by AS and any additional studies that did not to meet the inclusion criteria were excluded at this phase. Frequencies were utilized to describe nominal data while narrative analysis was carried out on the qualitative content to draw out the themes.

**Table 2:** General characteristics of included articles

References	Type	Geographic setting	Relevance to benefit sharing in biobanking
Árnason [24]	Report	Iceland	Highlights the ethical issues around the Icelandic biobank project (deCODE)
Berg [32]	Opinion Article	Ireland	Practical difficulties in implementing benefit sharing
Boggio et al. [45]	Book Chapter	Geneva	Comparative analysis of 27 biobanking policies on various ethical legal and social issues (ELSI)
Capron et al. [29]	Peer Reviewed Article—Empirical	27 countries	Ethical norms and the international governance of genetic databases and biobanks
Chalmers and Nicol [31]	E-book	Australia	Examines international best practice for the establishment, maintenance and use of human genetic research databases (HGRDs) and considers the measures that should be taken in Australia to comply with this best practice
Chen and Pang [48]	Peer Reviewed Article	Global	Discusses a fair, equitable and feasible biobank governance framework to ensure a fair balance of risks and benefits among all stakeholders
Emerson et al. [49]	Article-Debate	Not specified	Make a case for a tissue trust to respond to claims of exploitation through ‘scientific-imperialism’ and ‘bio-colonialism
Hobbs et al. [28]	Journal Article - Empirical	Germany and UK	Discusses appropriate methods of reciprocity in biobanking
Hugo ethics [38]	Opinion Article	N/A	Statement on benefit sharing by the Human Genome Organizations (HUGO) ethics committee
Joly et al. [47]	Peer Reviewed Article	N/A	Argues that open access can be considered benefit sharing in genomics research
Shickle (2014)	Book Section	N/A	Discusses various ethical legal and social issues (ELSI) in biobanking
Knoppers [39]	Peer Reviewed Article	Global	Discusses benefit sharing from the perspective of the Human Genome Organization’s (HUGO) ethics committee’s ‘Statement on Benefit-Sharing’
Laurie et al. [46]	Peer Reviewed Article	N/A	Attempts to reconcile individual privacy and public interests in genetic research using biobanks as a case
Mahomed et al. [35]	Peer Reviewed Article	South Africa	Examines issues surrounding transfer of human tissues across national boundaries and describes what a South African Institution considered for its material transfer agreements
Moodley et al. [26]	Peer Reviewed Article - Empirical	South Africa	Unearths research participants (of biobanking) concerns with storage of their tissue and use for research
National Bioethics Advisory Commission [23]	Report and Recommendations	USA	Report on research involving human biological materials in the USA. Discusses ethical issues and gives policy guidance
Ndebele and Musesengwa [43]	Peer Reviewed Article	Developing countries	Addresses the issue of fairness in benefits sharing and argues for justice in the sharing of both burdens and benefits of genetics research
Nicol and Critchley [27]	Peer Reviewed Article -Empirical	Australia	Discusses benefit sharing and biobanking in Australia
Pullman and Latus [44]	Peer Reviewed Article	N/A	Suggests some ways in which benefit-sharing might be implemented for genetic add-on studies
Ravinetto and Dierickx [33]	Peer Reviewed Article	India	Reviews relevance and applicability of benefit sharing in the revised “Indian National Ethical Guidelines for Biomedical and Health Research Involving Human Participants”
Schroeder and Gefenas [50]	Peer Reviewed Article	N/A	Examines post study obligations as a mechanism for benefit sharing
Schroeder and Lasen-Diaz [41]	Peer Reviewed Article	Global	Considers whether Convention on Biological Diversity (CBD) should be expanded to include human biological resources
Schroeder and Lucas [37]	Book Chapter	Global	Encourages further empirical research in order to move from theoretical understandings of fair benefit sharing to better practice which benefits real people
Schuklenk and Kleinsmidt [42]	Peer Reviewed Article	Global	Critically analyses benefit sharing looking at some of the practical challenges in sharing benefits such as Intellectual Property (IP) with communities
Sheremeta and Knoppers [40]	Peer Reviewed Article	Global	Discusses population genetics and benefit sharing
Simm [36]	Peer Reviewed Article	Global	Examines and clarifies the notion of benefit-sharing by focusing on its justifications
Vaz et al. [25]	Peer Reviewed Article-Empirical	India	Elicits views of ethics committee members and researchers involved in biobanking
Xiaoyong [30]	Book Chapter	China	Examines benefits sharing under different health research models
Yakubu et al. [34]	Peer Reviewed Article	South Africa	Highlights governance issues in biobanking

## Results

### Articles included in the scoping reviews

**Fig. 1:**
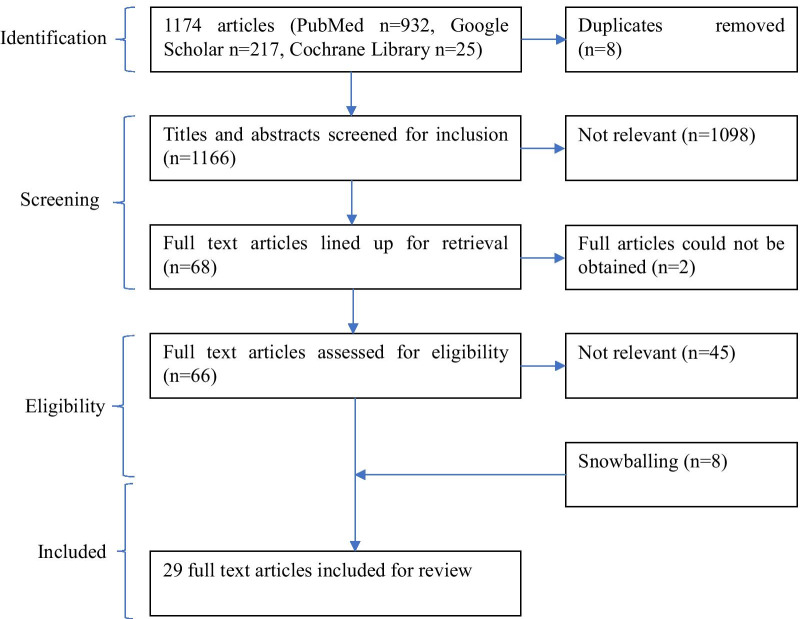
PRISMA Flow chart of study selection process

The search yielded 1174 potentially relevant citations. Eight duplicates were discarded after which the remaining citations were subjected to title and abstract relevance screening. At this stage a further 1098 citation were excluded. A total of 68 articles were lined up for retrieval of which 66 were eventually retrieved. Upon reading the full text, 21 articles met the inclusion criteria. An additional 8 articles were identified by going through the reference lists of the selected articles making the total number of articles included in the analysis 29 (see Fig. 1 below).

### General characteristics of included articles

Of all the articles included in the review, only one was published in 1999 [23] while the rest were published between 2000 and 2020. Journal articles formed majority of documents included in the review (22/29; 75.8%), followed by book chapters/sections (5/29; 17.2%). There were also 2 reports specific to benefit sharing that were included in the review [23, 24]. Five of the 29 documents were findings from empirical studies [25–29] while the rest were either reports, commentaries, opinions or debates. In terms of geographic settings for the empirical studies, there was one study each from India [25], Australia [27], South Africa [26] and UK/Germany [28]. One study was done across 27 different countries classified as 18 High, 6 Medium, and 3 Low-income [29]. Some of the non-empirical articles were also country-specific, and included one each from China [30], Australia [31], Ireland [32], India [33] and two from South Africa [34, 35]. The two reports included in the review were from the United States [23] and Iceland [24]. Majority of the articles discussed benefit sharing from a global perspective, often comparing high-income and low-income countries. Less than half of the documents reviewed (12/29; 41.4%) addressed benefit sharing as a stand-alone subject [30, 32, 33, 36–44] while the rest discussed it as one of several ethical issues in biobanking. While most of the articles looked at biobanking in general, 11 documents (37.9%) discussed biobanking within genetics/genomics research.

### Reported definition of benefits and benefit sharing

There were broad variation in definitions of the terms ‘benefit’ and ‘benefit sharing’ in the articles included in the review. Only three of the 29 articles specifically defined the term benefit [36, 40, 45]; all three made reference to the 2000 statement on benefit-sharing by the Human Genome Organization (HUGO) ethics committee that defined benefit as “a good that contributes to the wellbeing of an individual and/or a given community” [38]. A larger number of articles (n = 12) provided some form of definition or description of benefit sharing. Of those, 7 articles adopted, with slight variations, Schroeder et al.’s definition [11] earlier described [33, 37, 41, 43–46]. A further two articles listed the elements of the HUGO ethics committee statement on benefit sharing in providing their definitions [36, 40]. Three papers gave independent definitions of benefit sharing as “an equitable exchange in return for genetic resources” [47]; as a “process or action of sharing in the benefits that derive from the research in a manner that is fair and equitable” [35]; or as”mechanisms that might be put in place to ensure that benefits stemming from biobanking and use of biobank resources in biomedical research are not perceived to be the exclusive domain of the commercial sector” [27].

### Reported benefits

The reviewed articles describe a wide range of benefits in biobanking spanning those that can be enjoyed at individual level, at community level and at a global scale. Table 3 below highlights the types of benefits mentioned within the articles and provides some specific examples.

**Table 3:** Benefits and potential beneficiaries

Benefit	Example(s)	Beneficiaries	Articles
Financial	Licensing fees, percentage of profits, annual fees, direct payments	State, pharmaceutical companies, general population, researchers	[24–27, 29, 32–35, 38, 40, 44–48, 50]
Capacity building	Improvement of healthcare infrastructure (databases, physical infrastructure, research infrastructure), research staff training and development, technology transfer, and joint ventures	Public health facilities, research staff, research facilities, communities that utilize improved capacity	[24, 29, 35, 38–50]
Treatment or Healthcare	Free medical service (medication or consultation), provision of vaccines, tests, drugs, and treatments, treatment for non-research-related conditions	Research participants, their families, communities	[24–26, 28, 30, 32, 33, 37, 38, 40, 45–48, 50]
Improved understanding, knowledge or therapies	Improved understanding/new insights of disease processes and a potential for new therapeutic modalities	Whole of mankind, researchers, pharmaceutical industry, individuals, communities, and populations from which they are derived	[25, 32, 35, 38–40, 46–48, 50]
Post study access	Provision of pharmaceutical and diagnostics products that emerge from the research, giving access to new genetic tests, access to interventions identified as beneficial or to other appropriate care or benefits at subsidized cost	Research participants/specimen donors and their communities	[24–26, 29, 33, 37, 40, 45, 47, 50]
Return of results/findings	Provision of individual results (information about own health, incidental findings) or aggregate findings (general research results, study outcome) from the use of their samples	Specimen donors, their families/communities, research community	[26, 28, 33, 40, 45, 46]
Intellectual property/royalties	Publication rights, royalties from intellectual property, recognition	Researchers, pharmaceutical industry	[39, 40, 42, 45, 48]
Humanitarian efforts	Donation of percentage of profits/royalties for humanitarian efforts such as general education or health campaigns, community development projects (schools, clean water, roads), support mechanisms for destitute community members, insurance	Communities participating in biobanking	[24, 38, 40, 42, 50]
Responsiveness to local needs	Diagnostic or therapeutic application of the research that are tailored to local health needs	Communities where samples are collected/research is conducted	[40, 48, 50]
Jobs	Jobs and related economic activities generated by the research industry such as employment within research facilities and/or biobanks. Scientists and other cadre of staff	Local community members, local researchers and professionals	[24, 44]
Compensation for costs	Reimbursement of individual’s time, inconvenience and expenses	Research participants/specimen donors	[38, 39]
Counselling, screening services and testing	Genetic counselling, screening tests, free tests, regular health checks	Specimen donors, their families	[28, 45]
Other benefits	Anything can be shared, as long as it is defined as a benefit by a substantial number of stakeholders. Even recognition of participants contribution and thanking them	Different stakeholders involved in biobanking	[24, 36, 38]

### Justification for benefit sharing

In the papers reviewed, a significant amount (18/29; 62.1%) justify the importance of benefit sharing in biobanking, with the majority (13/18; 72.2%) indicating that benefit sharing is important as a matter of justice and as a means of redressing existing inequalities, promoting fairness and equality and addressing potential for exploitation [25, 32, 33, 35–37, 39, 42, 43, 47–50]. Other reasons stated included sharing benefits as a moral duty/ethical obligation [25, 33, 39, 44, 45, 49]; in order to comply with existing regulation/ethical principles on benefit sharing [27, 39, 42, 43]; that we share a common heritage and therefore benefits should be shared in solidarity/or as a common good [27, 39, 40]; to respond to participant needs/requests [39, 48]; and that investments by private enterprises currently exceed contributions by governments and that the enterprises need to share the benefits [36, 38, 41].

### Governance mechanisms for benefit sharing

Of the documents we reviewed majority (22/29; 75.9%) mention ways in which benefit sharing can be governed in biobanking. The governance mechanisms range from institutional level policies to international level guidelines and frameworks. A summary of the mechanisms, relevant clauses and articles that make reference to governance is presented in Table 4 below.

**Table 4:** Governance mechanisms for benefit sharing

Governance mechanism			Relevant provisions	Articles
1	Convention on Biological Diversity	(a) Bonn Guidelines on Access to Genetic Resources and Fair and Equitable Sharing of the Benefits Arising out of their Utilization[51]	Both monetary (e.g. access fees, upfront payment, joint ownership of relevant IP rights) and non-monetary (e.g. sharing of research and development results, collaboration, development programs to build local capacity) benefits ought to be shared	[41, 42, 46, 49]
		(b) The Nagoya Protocol on Access and Benefit-sharing [52]	Various provisions on the conservation of biological diversity, the sustainable use of its components and the fair and equitable sharing of the benefits arising out of their utilization	[24, 30, 37, 40–42, 45, 47]
2	UNESCO	(a) Universal Declaration on the Human Genome and Human Rights[53]	Benefits from advances in biology, genetics and medicine, concerning the human genome, shall be made available to all, with due regard for the dignity and human rights of each individual	[24, 27, 31, 39, 40, 45–48]
		(b) Universal Declaration on Bioethics and Human Rights 2005[54]	Benefits should be shared with society as a whole, within the international community and with developing countries. Such benefits include “special and sustainable assistance to, and acknowledgement of, the persons and groups that have taken part in the research”	[24, 27, 33, 37, 41, 43, 46, 50]
3	HUGO statement on benefit sharing [38]		Undue inducement for human genetic samples through compensation ought to be prohibited	[24, 27, 40–44, 47–49]
			Technology transfer, local training, joint ventures, health care provision, infrastructure provision, payment of expenses, and the use of royalties for humanitarian purposes ought to be encouraged	
			1–3% of net profits be donated to local, national and international humanitarian efforts	
4	The Nuffield Council report on Human Tissue Ethical and Legal Issues [55]		Discussions on the link between commercialization and benefit sharing: i.e. property rights over the actual tissue, claims of entitlement to share in any benefits arising from the exploitation of the tissue and, any consequent intellectual property rights	[45]
5	WHO guidelines	(a) International Guidelines on Ethical Issues in Medical Genetics and Genetic Services [56]	Families or ethnic groups with a particular variant or disease, whose genetic information results in a patent, should receive some benefit in return	[39, 45, 50]
		(b) European partnership on patients’ rights and citizens’ empowerment [57]	Some kind of benefit will ultimately be returned, either to the individual from who the materials were taken, or to the general class of person to which that individual belongs	
6	Council for International Organisations of the Medical Sciences (CIOMS) guidelines[58]		Give priority to direct benefits over indirect benefits	[33, 43, 45, 50]
			All research in developing countries and sponsored by developed countries, should be of relevance to the developing countries	
7	OECD Guidelines for Human Biobanks and Genetic Research Databases[59]		Sharing of knowledge is an important benefit to be derived from human biobanks and genetic research databases (HBGRDs)	[27, 46, 48]
8	UN Declaration on Human Rights[60]		Everyone has the right freely to “share in scientific advancement and its benefits”	[46]
9	The Declaration of Helsinki[61]		At the end of any research study, every subject entered in the project should be assured of the best proven prophylactic, diagnostic and therapeutic methods identified by that study	[33, 37, 43, 50]
10	Governmental laws, policies and regulations		Countries specific laws, policies and regulations including:	[24, 30, 31, 34, 35, 37, 39, 42, 43, 46, 48, 50]
			(a) Mandating return of benefits to participants and families	
			(b) Regulations on proprietary claims in respect of human tissue	
			(c) Laws on apportionment of profits or tools for benefit-sharing	
			(d) Statutory categories of benefit	
11	Ethics committees and regulatory bodies		(a) Checking for presence of benefit sharing provisions in protocols	[25, 33, 35, 37, 39, 43, 44, 46, 50]
			(b) Ensuring communities and individuals are not exploited	
			(c) Ensuring commercially exploitable projects make considerations for benefit-sharing	
			(d) Approving benefit-sharing proposals presented to them	
			(e) Providing guidance on who should provide benefits in order to ensure compliance	
12	Institutional policies and frameworks for benefit sharing		(a) Intellectual property policies	[25, 33, 40, 42, 44]
			(b) Information to be included in ICFs	
			(c) Requirements for benefit sharing agreements	
			(d) Return of results and compensation of participants	
13	Benefit sharing agreements		Specifies under which conditions benefits are to be shared, which benefits to be shared and proportion of sharing. Can exist at different levels:	[25, 29, 32, 41, 49]
			(a) International level e.g. conventions between governments and industry players	
			(b) Between industry and researchers	
			(c) Between biobanks and researchers	
			(d) Between/among biobanks	
			(e) Between all above players and research participants/specimen donors	
14	Material transfer agreements (MTAs)		Stipulates ethico‐legal requirements regarding the transfer of human biological materials	[35]

### Challenges to benefit sharing

A wide variety of conceptual and practical challenges to benefit sharing in biobanking were highlighted in 24 of the articles reviewed (see Table 5 for a summary). These range from the long periods associated with the development of any meaningful interventions from biobanking, the difficulties in determining who is to benefit and the proportions of those benefits, and the often large numbers of stakeholders involved (from specimen donors to researchers in multiple countries) and the power dynamics between them [24, 25, 28, 32, 33, 35, 37, 39–41, 44–47, 50]. Apart from benefit sharing in biobanking often being described as impracticable, articles also described challenges relating to cost with concerns that implementing benefit sharing would inflate the cost of research thereby discouraging investment especially in research that may not have big or immediate returns [28, 29, 31, 32, 37, 39, 50]. Another barrier mentioned in 12 articles is the lack of regulatory support/infrastructure for benefit sharing, specifically the lack of details in international and national ethics and guidance documents to guide implementation of benefit-sharing agreements. Institutions involved in biobanking are therefore left to interpret and implement benefit sharing as they deem best [24, 32, 33, 35, 37, 39, 42, 43, 45, 46, 48, 50]. Other issues identified included tensions between benefit sharing and other ethical principles such as coercion, undermining altruism and concerns about the commodification of human tissues.

**Table 5:** Benefit sharing challenges in biobanking

Challenge	Examples	Documents cited
Tensions with other ethical principles	(a) Payment or compensation of research participants/specimen donors: may be in conflict with conventions or guidelines that state that the human body should not be a source of income (commodification/commercialization of human body). Payments may also be considered coercing individuals to participate therefore a form of undue influence/coercion	[24–26, 29–33, 36–38, 41, 43–45, 50]
	(b) Claims of ownership of samples: It becomes difficult to distribute benefits when it is unclear who owns the samples	
	(c) De-identification of samples (individuals and communities): makes it difficult to share benefits with donors or return results if they are unknown	
	(d) Providing healthcare as a benefit: May be seen as undue inducement if provided to vulnerable individuals who have no other means of accessing care. Also brings up question of whose responsibility it is to provide care; researcher or government?	
	(e) Conflict between protection against undue inducement on the one hand and exploitation on the other	
	(f) Conflict between business enterprise required to fund research and claims of commercialization of human body in the process	
Practicality Issues	(a) Long periods between tissue collection and development of interventions: benefit not immediate or apparent	[24, 25, 28, 32, 33, 35, 37, 39–41, 44–47, 50]
	(b) Low yield: numerous attempts before successful intervention	
	(c) Nature of research: Samples may be used for basic research where no intervention is developed. Benefits such as post study access therefore become impractical	
	(d) Absence of royalties, profits and patents: Difficult to distribute benefits	
	(e) Nature of sample collection: Small amounts of tissue may be collected over wide geographical regions. If and when an intervention is developed, it may be difficult to share benefits among all	
	(f) Oversight: Due to long periods between sample collection and development of intervention (sometimes decades) it becomes difficult for Ethics committees or governments to perform oversight of benefit sharing	
	(g) Uncertainty: Not possible to tell how samples will be used, what interventions will be developed and therefore what benefits may accrue	
Weak governance	(a) No requirement for benefit sharing in legislation nor enforcement mechanisms	[24, 32, 33, 35, 37, 39, 42, 43, 45, 46, 48, 50]
	(b) No protections for poor countries from exploitation by richer countries	
	(c) Guidelines do not describe which benefits shall be shared and how benefit sharing would work practically	
	(d) Lack of clarity in guidelines on whether direct or indirect benefits should be shared	
	(e) Poor or absent medical and patents laws and/or regulatory frameworks in most countries	
	(f) Organizations and policies provide inconsistent and incomplete frameworks, and none of them possess supra-national status, authority or enforceability	
	(g) Laws prohibiting sale of tissues may work against compensation of sample donors (misconstrued as payment)	
	(h) Non-committal language in legislation e.g. “may”, “could be considered”	
	(i) Non-reliance by states of international declarations e.g. Declaration of Helsinki by the United States of America	
	(j) Inability by ethics committees to enforce benefit sharing requirements	
	(k) Narrow focus on one kind of benefit e.g. post study obligations by international guidance documents precludes other benefits	
	(l) Explicit exclusion of human biological materials from CBD	
	(m) No means for redressing past injustices e.g. samples already shipped out	
	(n) Precedence provided by some court rulings in which specimen donors have been denied right to share in benefits from their genetic materials	
Not current practice	(a) Superseded by other principles e.g. privacy, consent	[24, 27, 31–33, 35, 37, 39, 44, 50]
	(b) No ethical precedence/ long-standing ethical tradition for paying/compensating donors of biological material	
	(c) Attitude: Seen as unworkable or idealist by detractors; even questioning the need for benefit sharing	
	(d) Scant attention to governance of benefit sharing	
	(e) Presumption that tissue donation is purely altruistic	
	(f) Benefit sharing is still poorly understood and implemented, including by many key research stakeholders, such as researchers, sponsors, regulators and, sometimes, ethics committees	
	(g) Guidelines, checklists and templates from most ECs and Institutional Review Boards (IRBs) do not include “benefit sharing” among the issues to be checked/reviewed	
	(h) Negotiations about benefit sharing not a part of informed consent processes	
Undermining altruism	Focus on the sharing of financial benefits could attenuate people’s willingness to participate for idealistic reasons	[25, 26, 29, 31, 32, 36, 37, 45, 47]
Expensive	Implementing benefit sharing could increase cost of doing biobanking and research	[8, 28, 29, 31, 37, 39, 50]
Insufficient evidence to support/justify benefit sharing	(a) No empirical studies to demonstrate need for and how to execute benefit sharing in biobanking	[26–28, 32, 35, 37]
	(b) Little empirical research on what types of benefit sharing arrangements members of the public may wish to see incorporated into biobank governance and regulatory frameworks	
	(c) Seems intuitive to compensate tissue donors when their samples lead to generation of revenue but there are no specific arguments for this	
	(d) Specific, strong arguments for financial compensation to individuals are hard to find	
Procedural issues	(a) Who negotiates for benefit sharing?	[30, 37, 40, 42]
	(b) How is community defined?	
	(c) How are representatives to negotiate benefit sharing selected given existing structures may be undemocratic or exclude certain members	
	(d) Need for inclusion of other interest groups e.g. religious leaders	
Difficulty in quantifying contributions	(a) Donors vs donors: Should donors whose specimen can be directly attributed to intervention be the ones to be compensated?	[30, 32–37, 44, 47, 50]
	(b) Virtually impossible to determine the relative importance of any one sample to the overall success of the study	
	(c) Researchers vs donors: Does the sample provided by the specimen donor have inherent value or is value created by what the researcher does?	
	(d) Researchers vs researchers: How should benefits shared between providing entity/biobank and recipient entity/biobank. Also, researchers from low-income countries compared to those from rich countries	

### Mechanisms for benefit sharing

A total of 11 articles we reviewed propose mechanisms for benefit sharing. Benefit sharing agreements—described as formal engagements between two or more parties involved in biobanking in which decisions about how benefits that arise in biobanking would be shared—were described in 5 articles [25, 29, 32, 41, 49]. However, they did not provide information on what such agreements should contain, and how they could be negotiated including who would be involved. Some of the articles proposed consultations with stakeholders at different stages of the biobanking process [38, 41, 47, 49]. However, apart from participants/ specimen donors and their communities, the articles do not describe which other parties should be consulted, the specific issues to be discussed, at what stage of the biobanking process nor the context under which the consultations should be undertaken.

The role of ethics/regulatory review in supporting benefit sharing was highlighted in 3 articles which discussed this role as limited to checking for specific benefit sharing provisions prior to approving primary research [33, 46, 50]. One of the articles proposed a tissue trust in which tissues are held in trust for the donors by a trustee who oversees uses in accordance with the wishes of the donors. The tissue trust is then capable of returning long term benefits to the source community that extend beyond the benefits of primary research, such as improvement of healthcare facilities and capacity building of local personnel [49].

## Discussion

In this paper, we set out to explore the considerations and practices for benefit sharing in biobanking. To guide our discussions, we began by describing the definitions of ‘benefits’ and ‘benefit sharing’ in the context of biobanking. The articles we reviewed characterised ‘benefits’ in biobanking very broadly to include anything that contributes to the wellbeing of an individual or community at local, national or global level. This broad definition allows for the inclusion of a wide variety of benefits ranging from provision of basic, tangible household supplies such as soap, to more complex and less tangible items such as building research skills within the community [14, 17]. Although this non-specificity in the description of benefits expands the breadth of what could be considered as a benefit in biobanking, it also further complicates considerations for benefit-sharing in biobanking due to its broad nature.

Similarly, among the reviewed articles, benefit sharing in biobanking was broadly described to encompass the fair and equitable distribution of any benefits that accrue from biobanking. Although it was considered an ethical obligation and discussed alongside other ethical issues in research such as consent, privacy and confidentiality, it did not receive specific attention as a stand-alone ethical issue within the reviewed articles. Whilst biobanking is a supportive function of research rather than research, benefit sharing in biobanking has mostly been framed in a similar manner to benefit sharing in health research.

The challenges to benefit sharing outlined in the reviewed papers are not unique to biobanking but are magnified by the nature of biobanking. Biobanking typically tends to blend primary research, which is organised around specific research questions and methods, with more open-ended secondary research which is often unknown at the time of sample collection. It is not always clear at the time of sample collection exactly what kind of secondary research will be done in the future with the samples, when, by whom, and what product/intervention might be realised, if any. This lack of clarity coupled with the fact that there may be long intervening periods between sample collection and intervention development, means that biobanking benefits are often not immediately known or apparent and raises important unresolved questions such as whether benefit sharing ought to be contingent on intervention development. The pooling of samples from different studies, communities or even biobanks as well as requirements for de-identification mean that it might be difficult to attribute inventions to particular communities thereby further complicating return of benefits to sample donors or their communities.

Majority of the challenges plaguing benefit sharing in biobanking seem to be those stemming from the uncertainties about if, when, by whom and for what purposes stored samples will be used and the attendant difficulty in predicting benefits. Additionally, benefit sharing in biobanking is still poorly understood with some stakeholders perceiving it as idealistic or unworkable and further confounded by weak governance mechanisms.

Benefit sharing ideally involves various stakeholders who have different roles as providers, producers, and recipients of benefits. Various stakeholders are described as important in benefits sharing arrangements in biobanking. These stakeholders include sample donors, their families/communities, researchers, regulatory bodies, biobanks, research institutions, countries and the global community. However, a number of areas remain unclear including which stakeholders play what role- if any, extent to which they are involved in benefit sharing discourse/negotiations, when and how benefit sharing decisions would be made and the regulatory environment within which such stakeholder interactions should take place.

The reviewed articles refer to a wide variety of governance mechanisms that can be drawn upon to guide benefit sharing in biobanking. However, most of the stated mechanisms are from related fields such as genomics, health research, human rights and other benefit sharing conventions. The lack of specificity to biobanking underscores the need to develop governance mechanisms that are not only targeted at biobanking, but also context-specific and have the potential for international application especially when samples and data are shared across borders.

## Limitations

Despite attempts to be as comprehensive as possible, this review only utilised free online databases to perform literature searches and may not have identified all relevant articles in the published and grey literature. Searching other bibliographic databases may have yielded additional literature. Similarly, our inclusion of articles published in English only constitutes a limitation to our analysis.

## Conclusions

Biobanking is expanding rapidly globally whilst benefit sharing in biobanking is still at its infancy. This is reflected in the limited number of dedicated articles and empirical studies in the review. Based on the literature reviewed, we highlight the main gaps in key areas informing benefit sharing in biobanking and draw attention to the need for empirical research involving various categories of stakeholders in both LMICs and HICs in order to: support how stakeholders define what counts as appropriate benefits for them; demonstrate the need for benefit sharing; clarify contentious issues such as under what circumstances benefits should be shared, in what form, with which stakeholders; and how decisions about benefit sharing should made.

Traditional benefit sharing mechanisms such as material transfer agreements and ethics or regulatory review may be insufficient in addressing the complex nature of benefit sharing in biobanking; due to their focus on discrete research protocols. Alternative, novel mechanisms such as benefit sharing agreements, that can be developed and used concurrently, at micro-level (between specimen donors/communities and researchers), meso-level (between researchers and biobanks or between biobanks) and macro levels (between countries) have been proposed. Such approaches may provide the latitude to account for the different benefit sharing levels, the needs of the stakeholders within particular settings and can include clauses for amendments, allowing for progressive decision making as more information becomes available during the biobanking cycle. However, there is paucity of empiric information on these and other benefit sharing mechanisms. Further research could help inform the development, adoption, implementation and testing of benefit sharing mechanisms appropriate for biobanking within different contexts.

## Supplementary Information

**Additional file 1. Appendix 1**: Title and Abstract screening tool.

**Additional file 2. Appendix 2**: Google Scholar and Cochrane Library search strings.

## Data Availability

All data generated or analysed during this study are included in this published article and its supplementary information files.

## Abbreviations

CBD: Convention on Biological Diversity
CIOMS: Council for International Organizations of Medical Sciences
ECs: Ethics Committees
ELSI: Ethical Legal and Social Issues
HGRDs: Human genetic research databases
HUGO: Human Genome Organizations
IP: Intellectual property
IRBs: Institutional Review Boards
KEMRI: Kenya Medical Research Institute
MeSH: Medical subject headings
N/A: Not applicable
OECD: Organisation for Economic Co-operation and Development
PRISMA: Preferred Reporting Items for Systematic Reviews and Meta-Analysis
UK: United Kingdom
UN: United Nations
UNESCO: United Nations Educational, Scientific and Cultural Organization
USA: United States of America
WHO: World Health Organization
