# Supplementary material for: A scoping review of considerations and practices for benefit sharing in biobanking
Source: BMC Med Ethics. 2021 Jul 27;22:102. doi: 10.1186/s12910-021-00671-x (PMC8317360; doi:10.1186/s12910-021-00671-x)
Supplement: Supplementary file 1 — Additional file 1. Appendix 1: Title and Abstract screening tool. [file 12910_2021_671_MOESM1_ESM.docx]

# Appendix 1: Title and Abstract screening tool

1. Does the citation (title or abstract) describe any aspect of biobanking or the MeSH term biological specimen bank?
   - - Yes,
     - No,
     - Can’t tell.
2. Does the citation (title or abstract) describe biobanking for research?
   - - Yes,
     - No,
     - Can’t tell.
3. Does the citation (title or abstract) describe benefit sharing among different stakeholders involved?
   - - Yes,
     - No,
     - Can’t tell

Reviewer Decision:

- If the reviewer answer is “No” to any of the questions, disqualify citation
- If the reviewer answer is “Yes” to all the questions, select article for retrieval /further screening and appraisal.
- If the reviewer answer is “Can’t tell” to one of the questions, select article for retrieval /further screening and appraisal.
