# Supplementary material for: A scoping review of considerations and practices for benefit sharing in biobanking
Source: BMC Med Ethics. 2021 Jul 27;22:102. doi: 10.1186/s12910-021-00671-x (PMC8317360; doi:10.1186/s12910-021-00671-x)
Supplement: Supplementary file 2 — Additional file 2. Appendix 2: Google Scholar and Cochrane Library search strings. [file 12910_2021_671_MOESM2_ESM.docx]

# Appendix 2: Google Scholar and Cochrane Library search strings

1. **Google Scholar**

**Initial search strings**

- - 1. allintitle: ("benefit sharing" OR "benefit distribution" OR "benefit allocation" OR "benefit division" OR "burden sharing" OR "burden distribution") AND (Biobank OR biorepository OR Specimen OR Bank OR Substance OR Tissue OR biological) -plant: 69 relevant articles
    2. ("benefit sharing" OR "benefit distribution" OR "benefit allocation" OR "benefit division") AND (Biobank OR biorepository OR Bank OR Tissue) AND (research)-biodiversity -fiscal -climate -nagoya -plant -water -wildlife -seed -forest: 145 relevant articles

**Search string comprising reviewer suggested terms**

- - 1. allintitle: (Benefit OR profit OR reward OR advantage OR gift OR money OR dividend) AND (sharing OR distribution OR allocation OR division OR payment OR recompense) AND (biobank OR biorepository OR bank OR tissue OR specimen OR biological) AND (research): 3 relevant articles

1. **Cochrane Library**

#1 MeSH descriptor: [Biological Specimen Banks] this term only 16

#2 (biobank):ti,ab,kw OR (biorepositor):ti,ab,kw 477

#3 MeSH descriptor: [Beneficence] explode all trees 2

#4 ("benefit sharing"):ti,ab,kw OR ("social value"):ti,ab,kw OR ("benefit distribution"):ti,ab,kw (Word variations have been searched) 203

#5 MeSH descriptor: [Risk Assessment] explode all trees 8752

#6 ("risk distribution"):ti,ab,kw OR (burden):ti,ab,kw 19747

#7 #1 OR #2 486

#8 #3 OR #4 204

#9 #5 OR #6 28313

#10 #7 AND #8 0

#11 #7 AND #9 25

#12 #10 OR #11 25
